# Prognostication of Mental Health Risk Clusters on Hospitalization and Mortality in Patients With Coexisting Diabetes and Kidney Failure: The Hidden Burden of Loneliness

**DOI:** 10.1016/j.xkme.2025.101099

**Published:** 2025-09-11

**Authors:** Rui She, Stanton P. Newman, Augustine Kang, Jason C.J. Choo, Erik Khoo, Mooppil Nandakumar, Konstadina Griva

**Affiliations:** 1 Department of Rehabilitation Sciences, the Hong Kong Polytechnic University, Hong Kong SAR, China; 2 Population and Global Health, Lee Kong Chian School of Medicine, Imperial College and Nanyang Technological University, Singapore City, Singapore; 3 City St George’s, University, University of London, UK; 4 Stanford University School of Medicine, Stanford, CA; 5 The National Kidney Foundation, Singapore; 6 Department of Renal Medicine, Singapore General Hospital, Singapore; 7 Yong Loo Lin School of Medicine, National University of Singapore, Singapore

**Keywords:** Comorbid condition, end-stage kidney disease, hospitalization, loneliness, mortality, psychosocial distress

## Abstract

**Rationale & Objective:**

Individuals with comorbid diabetes and kidney failure have poor clinical prognosis, often aggravated by psychological distress. Identifying individuals most at risk is crucial to improving service provision. This study aimed to identify psychosocial profiles in patients with diabetes and kidney failure, model their prognostic effects on hospitalization and mortality, and explore underlying mechanisms linking psychosocial health to clinical outcomes.

**Study Design:**

Prospective cohort study.

**Setting & Participants:**

A total of 221 participants with coexisting diabetes and kidney failure (median age: 59 years, 60.6% men) receiving hemodialysis were recruited from the National Kidney Foundation Singapore’s dialysis centers.

**Exposures:**

Depression, anxiety, loneliness, and hopelessness alongside self-care indicators were measured using validated self-reported scales.

**Outcomes:**

All-cause hospitalization and mortality were ascertained from medical records.

**Analytical Approach:**

Latent profile analysis was used to identify psychosocial profiles. Associations of sociodemographic, clinical factors and psychosocial profiles with clinical endpoints were modeled with Negative binomial and Cox regressions (mean = 21.8 months). Casual mediation analyses modeled self-care as mediator.

**Results:**

Three psychosocial profiles emerged: resilient (37.6%; all below cutoffs), overwhelmed (30.3%; above cutoffs), and lonely (32.1%; above cutoff for loneliness only). The lonely group was more socioeconomically disadvantaged relative to the resilient group. The lonely and overwhelmed groups had increased hospitalization rates and more hospitalization days than the resilient group (incident risk ratio [IRR] range, 1.50-1.82; *P* < 0.05). No association with mortality was found. Better diabetes self-care and nutrition quality-of-life also predicted hospitalization (IRR range, 0.94-0.97; *P* < 0.05) and mortality (hazard ratio [HR] = 0.93 and 0.96). Mediation analysis indicated that diabetes self-care activities accounted for 18% of the associations between the lonely profile and hospitalization days.

**Limitations:**

Geographic generalizability of participants and sample size.

**Conclusions:**

Interconnected psychosocial burdens significantly affect disease management and hospitalization risk in patients with diabetes and kidney failure. Integrating psychosocial screening and interventions into clinical practice, particularly addressing loneliness and not just depression and anxiety, may be crucial.

## Plain Language Summary

Patients with coexisting diabetes and kidney failure often struggle with emotional distress and complex treatments. Although depression and anxiety receive attention, loneliness and hopelessness are often overlooked. Our study explored how these psychosocial factors combine to affect self-care and clinical outcomes. We identified 3 distinct groups: resilient (low distress), lonely (high loneliness), and overwhelmed (severe distress). Both the lonely and overwhelmed groups showed significantly higher hospitalization rates than the resilient group. Poor diabetes self-care partly explained why lonely patients were hospitalized more often. These results emphasize the importance of screening for loneliness alongside traditional mental health concerns in kidney care. Targeted interventions to enhance self-management and social connections may improve outcomes for these vulnerable patients.

The escalating rates of chronic kidney disease (CKD) and kidney failure pose a substantial global health and health care burden for systems and providers at the point of care.^1^ Diabetes is one of the leading causes of kidney failure,^2^^,^^3^ and patients with comorbid diabetes and kidney failure constitute a particularly vulnerable segment of the population with kidney failure.^1^^,^^4^ They have poorer clinical prognosis (eg, greater risk of cardiovascular events and mortality) and higher health care utilization compared with nondiabetic patients with kidney failure and also those with nondialysis-dependent CKD.^5^^,^^6^

The management of comorbid diabetes and kidney failure is inherently complex because of multimorbidity, high symptom burden, onerous treatment regimens, and guidelines for the 2 conditions that are often in conflict (eg, diet).^7^ Depression is prevalent in patients with kidney failure. Pooled estimate rates range from 23% and 39% for clinical diagnostic interviews and rating scales, respectively.^8^ Elevated anxiety levels have also been reported by patients with comorbid diabetes and kidney failure compared with their non-DM counterparts.^4^ Depression has been associated with low self-care^9^ and increased hospitalization and mortality^10^^,^^11^ and, as such, has been the dominant focus in research and clinical practice. Other critical psychosocial risks, however, have received less attention in the context of CKD. Loneliness, the subjective experience of social disconnection, is an important health risk factor for all-cause and disease-specific mortality^12^ and has recently been linked to incident CKD risk.^13^ For patients with diabetes and kidney failure, the substantial treatment demands that interfere with valued activities, and physical limitations can precipitate social withdrawal and loneliness.^14^ The treatment demands are compounded by fear of treatment failure, helplessness, and uncertainty about the future, which in turn may foster hopelessness.^15^ Despite the interconnected psychological health risks in CKD, these are often studied in isolation, limiting our understanding of the psychosocial drivers of poor outcomes.

In addition, the mechanisms linking these psychosocial risk factors to adverse clinical outcomes are not well understood. Studies have consistently noted a significant association of depression with adherence markers^9^^,^^16^ and that poor self-care is associated with worse clinical outcomes;^16^^,^^17^ however, the potential mediating effect of self-care has not been empirically examined or modeled.

To address these knowledge gaps, this study aimed (1) to characterize the psychosocial profiles of patients with coexisting diabetes and kidney failure across multiple psychosocial risk indicators (ie, depression, anxiety, loneliness, hopelessness) using latent profile analysis; (2) to examine associations of these psychosocial profiles on self-care, hospitalization, and mortality; and (3) to model if self-care mediates the associations between psychosocial profiles and clinical outcomes.

## Methods

### Study Design

This prospective observational study involved patients recruited between October 2014 and July 2015 from the Combined Diabetes and Renal Control Trial, who were followed up to November 1, 2016.^18^ The Combined Diabetes and Renal Control Trial aimed to develop and evaluate an intervention for patients with diabetes and kidney failure, informed by a mixed-method observational study conducted as a needs assessment. The data presented here are part of this observational study, which focuses on outcomes such as self-care, all-cause hospitalization, and mortality.

Ethics approval was obtained from the National University of Singapore Institutional Review Board. All study participants provided written informed consent.

### Participants

Patients were recruited from the National Kidney Foundation Singapore’s dialysis centers. The inclusion criteria were as follows: (1) diagnosed with diabetes mellitus and on maintenance hemodialysis for more than 3 months; (2) proficiency in English, Mandarin, or Malay; and (3) absence of speech difficulties or severe cognitive impairment. Individuals with documented dementia-related diagnoses were excluded. Eligibility was verified through medical chart reviews conducted by nurse managers at each dialysis center.

### Measurements

#### Psychosocial Indicators

##### Depression and Anxiety

Hospital Anxiety and Depression Scale was used to assess the severity of anxious and depressive symptoms.^19^ Participants rated symptoms such as low mood, panic, and loss of interest within the past week on a 4-point Likert scale. Each subscale has 7 items, and the total scores range from 0 to 21, with higher scores indicating more severe symptoms. A cutoff value of ≥8 is commonly used to indicate the presence of probable depression or anxiety.^20^ Hospital Anxiety and Depression Scale has been well-validated in DM and kidney failure populations, as well as in the local context.^21^

##### Loneliness

The 8-item Short-form UCLA Loneliness Scale was used to measure feelings of loneliness and social isolation.^22^ Each item is rated on a 4-point Likert scale (“never” to “always”) with total scores ranging from 8 to 32. Higher scores indicated greater loneliness and a cutoff value of ≥17 was used to denote mild loneliness.^23^

##### Hopelessness

The short version of the Beck Hopelessness Scale was used to assess people’s negative expectations of the future as an indicator of suicide risk.^24^ Sample items include “My future seems dark to me.” Items are rated on a 4-point Likert scale, with higher scores indicating higher hopelessness. A clinical cutoff score of ≥6 was used to indicate suicide risk.^24^

#### Self-care Indicators

##### The Summary of Diabetes Self-Care Activities Revised Version

It evaluates various domains of diabetes self-care behaviors, including general diet, exercise, blood glucose testing, foot care, and medication.^25^ It calculates the average number of days per week each activity is performed, with higher total scores indicating better self-management.

##### Dialysis Diet and Fluid Nonadherence Questionnaire

This patient self-report instrument measures nonadherence to fluid and dietary guidelines specific to dialysis patients respectively, calculated by multiplying the frequency and degree of nonadherence.^26^

##### Morisky Medication Adherence Scale

This 8-item scale assesses medication adherence with a total score ranging from 0 to 8.^27^ Higher scores indicate better adherence.

##### Treatment Self-Regulation Questionnaire

This tool evaluates autonomous motivation and controlled motivation for taking medications and checking glucose (8 items) and for following dietary and fluid rules (11 items).^28^ Items are rated on a 7-point Likert-type scale ranging from 1 (not at all true) to 7 (very true).

##### The cognitive restraint subscale of Three Factor Eating Questionnaire

It measures control over food intake, with items rated on a 4-point scale, with higher values signifying greater restraint.^29^

##### Nutrition-Specific Quality-of-Life

This 15-item questionnaire, derived from the Appetite and Diet Assessment Tool and the Food Enjoyment in Dialysis tool, assesses appetite-related quality-of-life in patients receiving hemodialysis.^30^

#### Hospitalization and Mortality

Data on hospitalizations and deaths following study enrollment were collected from medical records and the National Kidney Foundation’s administrative database (mean follow-up duration: 21.8 months; range, 15.1-24.8). Each admission, including any within hospital transfers, was counted as one hospitalization event. The duration of hospitalization was calculated from admission to discharge, with same-day admissions and discharge counted as one day. Mortality data, including the time from study enrollment to death, were recorded. Patients who withdrew from the study early because of transplantation or other medical reasons were censored. Detailed information on reasons for hospitalization and deaths is available elsewhere.^7^

#### Sociodemographic and Clinical Characteristics

Sociodemographic data on age, sex (biological sex assigned at birth), ethnicity, marital status, education, employment, work ability, income, homeownership, housing, and living arrangements were self-reported. Clinical characteristics such as dialysis vintage and treatment regimen were extracted from medical records. The Charlson Comorbidity Index, a composite score based on 17 comorbid conditions and age, was used to quantify comorbid burden.^31^

### Statistical Analysis

Descriptive data were presented as means and standard deviations, or frequencies, where appropriate. Analyses were performed using SPSS, Mplus, and R with significance levels set at α = 0.05.

#### Latent Profile Analysis

Latent profile analysis, a robust mixture-model technique,^32^ was performed to identify homogeneous psychosocial risk classes based on standardized scores for depression, anxiety, loneliness, and hopelessness. Models of 1 to 6 profiles were estimated and compared on multiple indices and overall interpretation to determine the optimal number. Specifically, lower values of Akaike information criteria, Bayesian information criteria, and sample size-adjusted Bayesian information criteria indicate a better fitted model, whereas higher entropy values (≥0.8) suggest clear profile separation.^33^ The Lo–Mendell–Rubin likelihood ratio test and bootstrapped likelihood ratio test evaluated the relative model fit between *k* profile and *k*-1 profile models, with significant LRT *P* value indicating a better model fit for the *k* profile model.^33^

#### Regression Analysis

Multivariable linear regressions were employed to evaluate associations between psychosocial profiles and self-care outcomes, controlling for sociodemographic and clinical factors that were significant in univariate analyses (eg, age, employment, and Charlson Comorbidity Index). Unstandardized (B) and standardized coefficients (*β*) were reported, with *β* values of 0.10-0.29, 0.30-0.49, and 0.50 or greater indicating small, medium, and large effect sizes, respectively.^34^

Negative binomial regressions were conducted to identify predictors of hospitalization outcomes, accounting for the overdispersed nature of data.^35^ Sociodemographic and clinical variables associated with hospitalization outcomes in the univariate analysis were adjusted as covariates (eg, Charlson Comorbidity Index). An additional offset variable, namely, the natural logarithm of the time at risk, was included in the regression models. Time at risk was calculated by subtracting the cumulative number of hospitalized days from the time enrolled in the study, recognizing that currently hospitalized patients are not at risk for further hospitalization events.^36^ Incidence rate ratios (IRRs) and the corresponding 95% confidence intervals are reported.

Cox’s proportional hazards model was used to examine the mortality risk of patients with different psychosocial profiles and self-care statuses, adjusted for comorbid condition burden and homeownership, which were significantly associated with mortality in the univariate analyses. Survival times were calculated from study enrollment date to death or end of study follow-up, whichever occurred first. Patients who received a kidney transplant (N = 3) were censored. The proportional hazards assumption was evaluated by log-log plots and tested using Schoenfeld residuals with hazard ratios (HRs) reported.^37^

#### Mediation Analysis

Mediation hypotheses were tested using causal mediation analysis that decomposes the total effect of psychosocial distress profiles on hospitalization and mortality outcomes into natural direct and indirect effects. Only self-care mediators associated with both psychosocial profiles and clinical outcomes were considered, fulfilling the prerequisite criteria for mediation analysis. For each mediator, 2 regression models were estimated: (1) a linear regression model for the association between psychosocial profiles and self-care outcomes and (2) a negative binomial regression model for hospitalization outcomes, conditional on psychosocial profiles and the self-care mediator. Mediation analyses for mortality were not conducted because of the lack of significant associations between psychosocial profiles and mortality risk. Direct and indirect effects were estimated by combining parameter estimates from these models using bootstrapping with 2,000 iterations. The proportion mediated (indirect effect/total effect) was calculated to indicate the effect size of mediation.

## Results

### Study Participants

The mean age of the 221 participants was 59.0 years (standard deviation 9.8), 60.6% were males, 54.8% were Chinese, 52.5% attended secondary school or above, and 62.9% were married. The majority were unable to work (78.3%), had been receiving dialysis for more than 2 years (57.5%), and had comorbid conditions, such as diabetic eye disease (65.6%) and hypertension (89.1%).

### Selection of Latent Profiles

**Figure 1:**
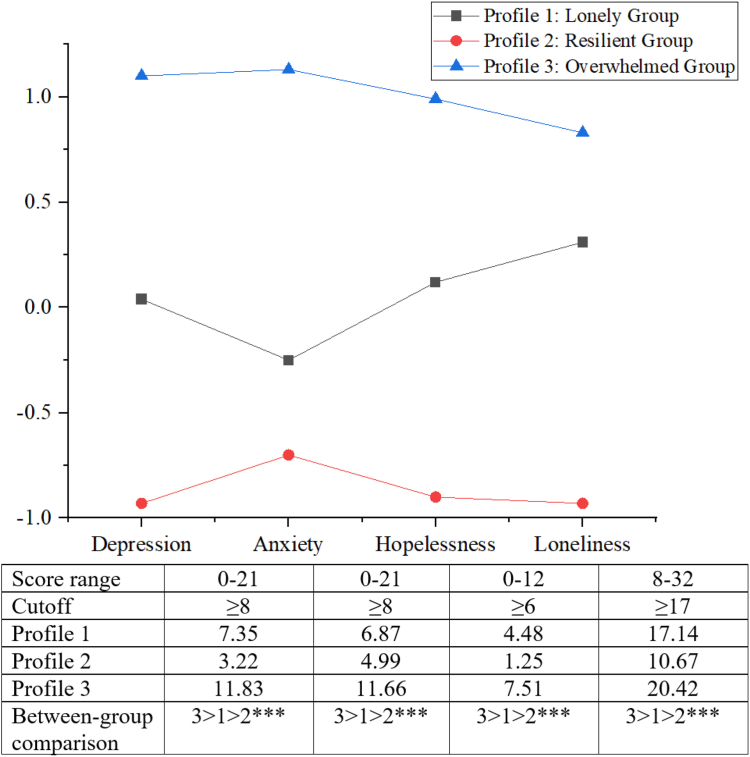
Description and standardized mean values of psychosocial indicators across profiles. Note: Between-group comparison in the levels of psychosocial indicators across profiles was conducted using one-way analysis of variance. ∗∗∗*P* < 0.001.

Table 1 displays model fit indices of latent profile analysis. Examination of Akaike information criteria, Bayesian information criteria, and sample size-adjusted Bayesian information criteria indicates that values decrease with the addition of a new profile in the models, and the curves of respective elbow plots of these indices flatten at the three-profile configuration (Fig S1). The Lo–Mendell–Rubin test also supported the 3-profile model over the 2-profile model, with non-significant improvements with increase in number of profiles. Although the 6-profile solution has the highest entropy, it included subgroups with small sample size (n < 20) and significant overlap in depression and anxiety indicators (Fig S2). In consideration of model parsimony, interpretability of profiles, and the proportion of individuals per profile (at least 5% to 10%), the 3-profile model was selected as the optimal solution. Figure 1 presents the profiles with standardized mean scores of psychosocial variables.

**Table 1:** Model Fit Indices for Latent Profile Analysis of Psychosocial Health

Number of Classes	AIC	BIC	aBIC	LMR-LRT	BLRT	Entropy	Sample Proportion (%) per Class
1				-	-	-	100.0
2	2,195.0	2,239.2	2,198.0	<0.001	<0.001	0.844	52.4/47.6
**3**	**2,123.4**	**2,184.6**	**2,127.5**	**0.008**	**<0.001**	**0.806**	**32.1/37.6/30.3**
4	2,103.6	2,181.7	2,108.8	0.083	<0.001	0.828	30.3/39.4/22.2/8.1
5	2,070.9	2,166.1	2,077.3	0.164	<0.001	0.843	28.1/23.1/26.7/14.9/7.2
6	2,046.8	2,158.9	2,054.3	0.093	<0.001	0.865	6.3/27.6/15.4/21.3/22.2/7.2

*Note:* The chosen three-class solution is highlighted in bold.

Abbreviations: AIC, Akaike information criterion; aBIC, sample size-adjusted BIC; BIC, Bayesian information criterion; BLRT, bootstrapped likelihood ratio test; LMR-LRT, Lo–Mendell–Rubin adjusted likelihood ratio test. Lower BIC and AIC values suggest a better fitting model and higher entropy values suggest that individuals are more distinctly classified into profiles. A significant LRT *P* value indicates that the model with *k* profiles fits the data significantly better than the model with *k*−1 profiles.

The 3 identified profiles comprised 32.1%, 37.6%, and 30.3% of the sample, respectively. Profile 1, labeled as “lonely,” was characterized by high levels of loneliness (mean values above the scale cutoff, see Fig 1), whereas depression, anxiety, and hopelessness were below cutoffs. Profile 2 was characterized as “Resilient” with the lowest values for all 4 psychosocial indicators (all z scores < –0.5 and below cutoffs). Profile 3, labeled “overwhelmed,” scored the highest on depression, anxiety, hopelessness, and loneliness (all z scores > 0.5 and above cutoffs). Between-group comparison using analysis of variance revealed that profile 3 exhibited the highest levels of psychosocial distress, followed by profile 1 and profile 2 (all *P* < 0.001).

### Interprofile Characteristic Differences

Demographic and clinical characteristics across profiles are detailed in Table 2. Compared with the resilient group, the lonely group were less likely to be married (OR = 0.27, *P* < 0.001) or homeowners (OR = 0.33, *P* < 0.01) and more likely to report being unable to work (OR = 4.48, *P* < 0.05). Those classified in the overwhelmed group were less likely to identify as Malay (OR = 0.48, *P* < 0.05), married (OR = 0.39, *P* < 0.01), and with higher rates of hypertension (OR = 4.39, *P* < 0.05), relative to the resilient group.

**Table 2:** Participants’ Characteristics and Differences by Latent Profiles Using Multinomial Logistic Regression

	Total Sample (n = 221)	Profile 1 (n = 71)	Profile 2 (n = 83)	Profile 3 (n = 67)	Profile 1 vs Profile 2	Profile 3 vs Profile 2	Profile 1 vs Profile 3
		Loneliness group	Resilient group	Overwhelmed group	OR (95% CI)	OR (95% CI)	OR (95% CI)
Sociodemographic Characteristics							
Age (y)	59.0 (9.8)	59.0 (9.3)	58.8 (8.6)	59.4 (11.6)	1.00 (0.97, 1.04)	1.01 (0.97, 1.04)	1.00 (0.96, 1.03)
Male	134 (60.6%)	40 (56.3%)	52 (62.7%)	42 (62.7%)	0.77 (0.40, 1.47)	1.00 (0.52, 1.95)	0.77 (0.39, 1.52)
Ethnicity							
Chinese	121 (54.8%)	41 (57.7%)	38 (45.8%)	42 (62.7%)	Reference	Reference	Reference
Malay	74 (33.5%)	22 (31.0%)	34 (41.0%)	18 (26.9%)	0.60 (0.30, 1.20)	**0.48 (0.23, 0.98)**^a^	1.25 (0.59, 2.67)
Others	26 (11.8%)	8 (11.3%)	11 (13.3%)	7 (10.4%)	0.67 (0.25, 1.85)	0.58 (0.20, 1.63)	1.17 (0.39, 3.52)
Marital status							
Not married	82 (37.1%)	36 (50.7%)	18 (21.7%)	28 (41.8%)	Reference	Reference	Reference
Married	139 (62.9%)	35 (49.3%)	65 (78.3%)	39 (58.2%)	**0.27 (0.13, 0.54)**^b^	**0.39 (0.19, 0.79)**^c^	0.70 (0.36, 1.37)
Educational levels							
Primary and below	105 (47.5%)	36 (50.7%)	38 (45.8%)	31 (46.3%)	Reference	Reference	Reference
Secondary	90 (40.7%)	25 (35.2%)	37 (44.6%)	28 (41.8%)	0.71 (0.36, 1.41)	0.93 (0.47, 1.84)	0.77 (0.37, 1.58)
Tertiary	26 (11.8%)	10 (14.1%)	8 (9.6%)	8 (11.9%)	1.32 (0.47, 3.72)	1.23 (0.41, 3.64)	1.08 (0.38, 3.06)
Employment status							
Employed	33 (14.9%)	8 (11.3%)	18 (21.7%)	7 (10.5%)	Reference	Reference	Reference
Unemployed	80 (36.2%)	31 (43.7%)	30 (36.1%)	19 (28.4%)	2.33 (0.88, 6.15)	1.63 (0.57, 4.63)	1.43 (0.45, 4.57)
Retired	45 (20.4%)	15 (21.1%)	16 (19.3%)	14 (20.9%)	2.11 (0.71, 6.28)	2.25 (0.73, 6.96)	0.94 (0.27, 3.27)
Family caregiver/others	63 (28.5%)	17 (23.9%)	19 (22.9%)	27 (40.4%)	2.01 (0.70, 5.81)	**3.65 (1.28, 10.46)**^a^	0.55 (0.17, 1.80)
Work ability							
Full-time work	23 (10.4%)	3 (4.2%)	13 (15.7%)	7 (10.4%)	Reference	Reference	Reference
Part-time work	25 (11.3%)	8 (11.3%)	12 (14.5%)	5 (7.5%)	2.89 (0.62, 13.50)	0.77 (0.19, 3.11)	3.73 (0.65, 21.58)
Unable to work and get paid	173 (78.3%)	60 (84.5%)	58 (69.9%)	55 (82.1%)	**4.48 (1.21, 16.55)**^a^	1.76 (0.65, 4.74)	2.55 (0.63, 10.33)
Monthly household income (Singapore dollar)							
$0–$2,000	109 (49.3%)	35 (49.3%)	37 (44.6%)	37 (55.2%)	Reference	Reference	Reference
$2,001 and above	71 (32.1%)	17 (23.9%)	30 (36.1%)	24 (35.8%)	0.60 (0.28, 1.27)	0.80 (0.40, 1.62)	0.75 (0.35, 1.62)
Do not know/do not wish to answer	41 (18.6%)	19 (26.8%)	16 (19.3%)	6 (9.0%)	1.26 (0.56, 2.82)	0.38 (0.13, 1.06)^d^	**3.35 (1.20, 9.35)**^a^
Homeownership (vs not)	137 (62.0%)	34 (47.9%)	61 (73.5%)	42 (62.7%)	**0.33 (0.17, 0.65)**^c^	0.61 (0.30, 1.21)	0.55 (0.28, 1.08)^d^
Housing^e^							
1-2 room HDB flat	31 (14.1%)	12 (16.9%)	11 (13.3%)	8 (12.1%)	Reference	Reference	Reference
3-4 room HDB flat	157 (71.4%)	49 (69.0%)	58 (69.9%)	50 (75.8%)	0.77 (0.31, 1.91)	1.19 (0.44, 3.18)	0.65 (0.25, 1.74)
5 room HDB and above	32 (14.5%)	10 (14.1%)	14 (16.9%)	8 (12.1%)	0.65 (0.21, 2.07)	0.79 (0.22, 2.77)	0.83 (0.23, 3.03)
Living with family (or not)	200 (90.5%)	62 (87.3%)	79 (95.2%)	59 (88.1%)	0.35 (0.10, 1.19)^d^	0.37 (0.11, 1.30)	0.93 (0.34, 2.58)
Disease and Treatment Status							
Age diagnosed with diabetes	38.5 (12.9)	39.0 (13.0)	37.9 (12.3)	38.8 (13.7)	1.01 (0.98, 1.03)	1.01 (0.98, 1.03)	1.00 (0.97, 1.03)
Time receiving dialysis^e^							
Less than 12 months	33 (15.0%)	12 (17.1%)	13 (15.7%)	8 (11.9%)	Reference	Reference	Reference
13 to 24 months	60 (27.3%)	19 (27.1%)	29 (34.9%)	12 (17.9%)	0.71 (0.27, 1.88)	0.67 (0.22, 2.03)	1.06 (0.33, 3.33)
More than 2 years	127 (57.7%)	39 (55.7%)	41 (49.4%)	47 (70.1%)	1.03 (0.42, 2.53)	1.86 (0.70, 4.94)	0.55 (0.21, 1.49)
Diagnosed diabetic eye disease							
No	66 (29.9%)	24 (33.8%)	21 (25.3%)	21 (31.3%)	Reference	Reference	Reference
Yes	145 (65.6%)	45 (63.4%)	56 (67.5%)	44 (65.7%)	0.70 (0.35, 1.43)	0.79 (0.38, 1.62)	0.89 (0.44, 1.83)
Do not know	10 (4.5%)	2 (2.8%)	6 (7.2%)	2 (3.0%)	0.29 (0.05, 1.60)	0.33 (0.06, 1.84)	0.87 (0.11, 6.77)
Diagnosed diabetic neuropathy in limbs							
No	142 (64.3%)	39 (54.9%)	55 (66.3%)	48 (71.6%)	Reference	Reference	Reference
Yes	71 (32.1%)	28 (39.4%)	27 (32.5%)	16 (23.9%)	1.46 (0.75, 2.86)	0.68 (0.33, 1.41)	**2.15 (1.02, 4.54)**^a^
Do not know	8 (3.6%)	4 (5.6%)	1 (1.2%)	3 (4.5%)	5.64 (0.61, 52.43)	3.44 (0.35, 34.15)	1.64 (0.35, 7.77)
Diagnosed high blood pressure	197 (89.1%)	65 (91.5%)	68 (82.9%)	64 (95.5%)	2.23 (0.81, 6.15)	**4.39 (1.21, 16.00)**^a^	0.51 (0.12, 2.12)
Charlson Comorbidity Index	9.20 (2.1)	9.10 (2.1)	9.23 (2.1)	9.25 (2.2)	0.97 (0.83, 1.13)	1.01 (0.87, 1.17)	0.97 (0.82, 1.13)

*Note:* Results with a significance level of *P* < 0.05 are highlighted in bold.

Abbreviations: CI, confidence interval; HDB, Housing and Development Board; OR, odds ratio.

a *P* < 0.05.

b *P* < 0.001.

c *P* < 0.01.

d 0.05 *<P* < 0.10.

e The sum does not equal the total sample size because of one missing case.

### Associations Between Psychosocial Profiles and Self-care

Both the lonely and overwhelmed groups reported significantly lower diabetes self-care activities (*β* = –0.20 and –0.19, respectively; all *P* < 0.05) and nutrition-specific quality-of-life (*β* = –0.39 and –0.38, respectively; all *P* < 0.001) compared with the resilient profile, adjusted for background covariates. The overwhelmed group also showed lower adherence to medication, diet, and fluid, less control over food, and poorer autonomous regulation of treatment than the resilient group (*β* range, –0.20 to –0.15; all *P* < 0.05) (Table 3).

**Table 3:** Associations of Psychosocial Health Profiles and Self-care Functioning (N = 221)

	Medication Adherence		Diet Nonadherence		Fluid Nonadherence		Diabetes Self-care Activities	
	*β*	B (95% CI)	*β*	B (95% CI)	*β*	B (95% CI)	*β*	B (95% CI)
Profile 2	0 (reference)		0 (reference)		0 (reference)		0 (reference)	
Profile 1	–0.06	–0.23 (–0.77, 0.32)	0.06	1.38 (–2.27, 5.03)	0.03	0.89 (–2.96, 4.74)	–0.20	–2.69 (–4.72, –0.66)^a^
Profile 3	–0.20	–0.76 (–1.31, –0.20)^a^	0.18	4.52 (0.81, 8.23)^b^	0.15	3.96 (0.04, 7.87)^b^	–0.19	–2.57 (–4.63, –0.52)^b^

	**Control over food intake**		**Autonomous regulation**		**Controlled regulation**		**Nutrition quality-of-life**	
	***β***	**B (95% CI)**	***β***	**B (95% CI)**	***β***	**B (95% CI)**	***β***	**B (95% CI)**
Profile 2	0 (reference)		0 (reference)		0 (reference)		0 (reference)	
Profile 1	–0.11	–0.37 (–0.86, 0.13)	–0.11	–0.23 (−0.53, 0.08)	–0.05	–0.16 (–0.61, 0.29)	–0.39	–3.62 (–4.96, –2.28)^c^
Profile 3	–0.16	–0.53 (–1.13, −0.03)^b^	–0.17	–0.35 (–0.66, –0.05)^b^	–0.10	–0.30 (–0.75, 0.16)	–0.38	–3.60 (–4.96, –2.25)^c^

Abbreviation: CI, confidence interval.

Controlled covariates included age, ethnicity, education levels, employment, marital status, and Charlson Comorbidity Index, which were significantly associated with self-care outcomes in the univariate analysis.

a *P* < 0.01.

b *P* < 0.05.

c *P* < 0.001.

### Associations of Psychosocial Profiles and Self-care With Hospitalization Outcomes

In the adjusted models, both the lonely (IRR = 1.50, *P* < 0.036; IRR = 1.74, *P* < 0.001) and overwhelmed groups (IRR = 1.82, *P* < 0.002; IRR = 1.82, *P* < 0.001) had significantly higher rates of hospitalization admissions and accumulated days of hospitalization compared with the resilient group (Table 4).

**Table 4:** Associations of Psychosocial Profiles and Self-care Functioning With Health Care Utilization and Mortality (N = 220)

	Frequency of Hospitalization		Days of Hospitalization		Mortality	
	IRR (95% CI)^a^	*P* Value	IRR (95% CI)^b^	*P* Value	HR (95% CI)^c^	*P* Value
Profiles of Psychosocial Health						
Resilient group	1.00 (reference)		1.00 (reference)		1.00 (reference)	
Lonely group	1.50 (1.03, 2.18)	0.036^d^	1.74 (1.24, 2.44)	0.001^e^	0.89 (0.28, 2.83)	0.850
Overwhelmed group	1.82 (1.25, 2.65)	0.002^e^	1.82 (1.30, 2.70)	<0.001^f^	1.65 (0.62, 4.40)	0.315
Self-care Ability						
Control over food intake (TFEQ)	0.91 (0.83, 1.01)	0.078	0.94 (0.87, 1.03)	0.203	0.72 (0.57, 0.91)	0.006^e^
Nonadherence to diet guidelines	1.01 (1.00, 1.02)	0.129	1.01 (1.00, 1.03)	0.099	1.02 (0.99, 1.05)	0.280
Nonadherence to fluid guidelines	1.01 (1.00, 1.03)	0.042^d^	1.02 (1.00, 1.03)	0.017^d^	1.02 (0.98, 1.05)	0.337
Diabetes self-care activities	0.97 (0.95, 1.00)	0.022^d^	0.96 (0.94, 0.99)	0.003^e^	0.93 (0.87, 1.00)	0.047^d^
Medication adherence	1.02 (0.93, 1.11)	0.694	0.99 (0.91, 1.07)	0.723	0.76 (0.60, 0.94)	0.014^d^
Autonomous treatment regulation	0.97 (0.83, 1.13)	0.675	1.07 (0.93, 1.24)	0.348	0.79 (0.52, 1.21)	0.283
Controlled treatment regulation	1.04 (0.92, 1.17)	0.539	1.08 (0.96, 1.21)	0.229	0.84 (0.61, 1.17)	0.302
Nutrition quality-of-life	0.96 (0.92, 0.99)	0.012^d^	0.94 (0.91, 0.97)	<0.001^f^	0.96 (0.92, 0.99)	0.012^d^

TFEQ, Three Factor Eating Questionnaire.

a Adjusted for homeownership and Charlson Comorbidity Index, which were significantly associated with the frequency of hospitalization in the univariate analysis.

b Adjusted for household income, housing arrangement, ethnicity, employment, living with family, and Charlson Comorbidity Index, which were significantly associated with the cumulative number of hospitalization days in the univariate analysis.

c Adjusted for age and Charlson Comorbidity Index, which were significantly associated with mortality in the univariate analysis.

d *P* < 0.05.

e *P* < 0.01.

f *P* < 0.001.

Better diabetes self-care and nutrition quality-of-life were associated with lower hospitalization rates and hospitalization days (IRR range, 0.94-0.97; all *P* < 0.05), whereas nonadherence to dialysis fluid guidelines were associated with increased higher hospitalization rates (IRR = 1.01, *P* = 0.042) and hospitalization days (IRR = 1.02, *P* = 0.017).

### Associations of Psychosocial Profiles and Self-care With Mortality

During follow-up, there were 23 deaths (10.4%). No significant associations were found between psychosocial profiles and mortality. However, better control over food intake (HR = 0.72, *P* = 0.006), diabetes self-care activities (HR = 0.93, *P* = 0.047), medication adherence (HR = 0.76, *P* = 0.014), and nutrition-specific quality-of-life (HR = 0.96, *P* = 0.012) predicted a low risk of death in the adjusted models (Table 4).

### Casual Mediation Analysis

Two self-care indicators, ie, diabetes self-care and nutrition quality-of-life that were significantly associated with psychosocial profiles and hospitalization outcomes were tested in mediation models. Results showed that diabetes self-care activities mediated the association between lonely versus resilient profiles and accumulated days of hospitalization (indirect effect: 2.30; 95% CI 0.01, 5.59), accounting for 17.8% of the total effect (Table 5). However, the meditation effect of diabetes self-care was not significant for the overwhelmed group, which instead showed a significant direct effect on hospitalization days (direct effect: 18.30; 95% CI: 5.23, 34.49). Additionally, no significant mediation effects were found for nutrition quality-of-life, nor were there significant mediation effects for the frequency of hospitalization events (v 5).

**Table 5:** Casual Mediation Analysis of Self-Care Functioning in the Association Between Psychosocial Profiles and Hospitalization Outcomes

	Frequency of Hospitalization^a^			Accumulated Days of Hospitalization^b^		
	Direct Effect (95% CI)	Indirect Effect (95% CI)	Direct Effect (95% CI)	Indirect Effect (95% CI)	Direct Effect (95% CI)	Indirect Effect (95% CI)
M1: Diabetes self-care						
Lonely vs resilient group	0.99 (–0.11, 2.13)^c^	0.12 (–0.12, 0.39)	-	10.65 (–1.28, 24.13)^c^	2.30 (0.01, 5.59)^d^	17.8%
Overwhelmed vs resilient group	1.87 (0.49, 3.27)^e^	0.11 (–0.10, 0.35)	-	18.30 (5.23, 34.49)^e^	2.04 (–0.46, 5.53)	10.0%
M2: Nutrition quality-of-life						
Lonely vs resilient group	0.85 (–0.45, 2.17)	0.21 (–0.25, 0.75)	-	9.10 (–7.22, 25.47)	3.80 (–0.97, 11.17)	-
Overwhelmed vs resilient group	1.68 (0.15, 3.33)^d^	0.22 (−0.23, 0.84)	-	12.35 (–3.36, 30.04)	4.03 (–1.23, 13.56)	-

Note: Direct effect: psychosocial profile → hospitalization; Indirect effect: psychosocial profile → self-care ability → hospitalization; Total effect: combined natural direct and indirect effect. Bootstrapping (n = 2,000) was used to estimate the indirect, direct, and total effects and the corresponding 95% confidence interval (CI).

-: Proportion mediated was not calculated for non-significant indirect effect.

a Adjusted for homeownership and Charlson Comorbidity Index.

b Adjusted for household income, housing arrangement, homeowner, ethnicity, employment, living with family, and Charlson Comorbidity Index.

c 0.05*< P* < 0.01.

d *P* < 0.05.

e *P* < 0.01.

## Discussion

To our knowledge, this is the first study to comprehensively characterize the psychosocial health status of patients with diabetes and kidney failure using multiple indicators (depression, anxiety, loneliness, and hopelessness) in relation to clinical endpoints and to examine the underlying mechanisms involved. We identified 3 distinct psychosocial profiles, each constituting approximately one-third of the sample: “resilient” (low in all distress indicators), “lonely” (characterized by high loneliness), and “overwhelmed” (characterized by co-occurring high levels of depression, anxiety, loneliness, and hopelessness). Notably, both the lonely and overwhelmed profiles were associated with increased risks of hospitalization over the ensuing 12 months compared with the resilient profile, with loneliness-related risks partially mediated by lower diabetes self-care activities.

There was sociodemographic and clinical heterogeneity across different psychosocial profiles. The lonely group was notably more socioeconomically disadvantaged than the resilient group, being single, nonhomeowners, and unable to work. The financial burden of hemodialysis is a significant stressor for patients with kidney failure in Singapore, as kidney replacement therapy is fee-for-service, and subsidies are not universal.^38^ Our findings indicated that many of the sample could not remain gainfully employed, with the greatest proportion in the “lonely” group. These challenges can lead to feelings of inferiority and lower self-esteem^39^ and may limit social engagement, thereby increasing the risk of social isolation and loneliness. The higher rates of unmarried status and comorbid conditions (eg, hypertension and diabetic neuropathy) in the lonely and overwhelmed groups further highlight the importance of familial support in maintaining mental well-being and the necessity of integrated care in managing coexisting diabetes and kidney failure.^40^ Regular mental health screening and proactive management of comorbid conditions (eg, pharmacist-led medication synchronization) may be incorporated into routine clinical visits. Furthermore, establishing supportive networks involving health care professionals, family, friends, community organizations, and volunteers is crucial for these vulnerable subgroups to alleviate the compound burden of mental distress and comorbid condition management.

The findings of increased hospitalization risk and poorer self-care among those classified in lonely and overwhelmed profiles are in line with prior studies on depression and anxiety in patients with kidney failure.^10^^,^^11^^,^^16^ Specifically, the probability of hospitalization during the follow-up period was 50% higher for the lonely group and 82% higher for the overwhelmed group, compared with the resilient group. As for hospitalization days, the magnitude of increased risk was similarly elevated for both groups (74% and 82%). Importantly, these associations were independent of sociodemographic and clinical factors (eg, comorbid condition burden). No associations were found between the psychosocial profiles and mortality, likely because of the small number of deaths in our sample, making it challenging to detect such associations.

The robust associations between the overwhelmed profile and various aspects of lower self-care further highlight the crucial role of emotional adjustment in chronic disease self-management, with small-to-moderate effect sizes.^41^ High distress may impair the cognitive capacity for self-care and motivation to engage with treatment regimens. In contrast, loneliness was primarily linked to poorer diabetes self-care and nutrition-related quality-of-life, likely because of low instrumental and emotional support for diet and glucose monitoring. Psychosocial distress, including loneliness, may further diminish dietary control and nutrition quality-of-life by altering appetite and affective experience with eating.^42^

In addition to psychosocial risk factors, better self-care, such as diabetes self-care and nutrition quality-of-life, was consistently associated with reduced risks of hospitalization and mortality. Improved self-care may signal better health literacy and self-efficacy in disease management and can translate into substantial benefits, such as early detection of symptoms, lifestyle modification, prevention of adverse events, and adherence and optimization of treatments, ultimately mitigating clinical risks.^43^ Notably, our study revealed that poorer diabetes self-care mediated the risk of longer hospitalization days in patients with the lonely profile relative to those with the resilient profile. Diabetes self-care involves multi-dimensional tasks, including diet, exercise, glucose monitoring, foot care, and medication adherence—all of which rely on supportive resources and are particularly vulnerable to social isolation.^44^ This also aligns with Leventhal’s Common-Sense Model of Self-Regulation, which posits that patients’ perceptions of their illness, including emotional distress and concerns resulting from chronic diseases, guide their self-management behaviors and decisions, ultimately affecting their illness outcomes.^45^ In contrast, this mediation effect was not observed for the overwhelmed profile, suggesting distinct mechanisms underlying the impacts of lonely and overwhelmed profiles on hospitalization. Biological mechanisms, such as stress-induced inflammatory pathways known to exacerbate physical disease progression,^46^ may help explain the increased hospitalization risk associated with the overwhelmed profile. Additionally, this group’s propensity for crisis-level distress might trigger emergency visits during psychosocial crises, a pathway less dependent on self-care activities.

Our study is the first to highlight the harm related to loneliness in the context of kidney failure. Although depression and anxiety have often been the focus of health interventions and clinical/psychological services, our study underscores that loneliness, regardless of co-occurring depression and anxiety, increases hospitalization risk. Addressing loneliness is thus important for improving patient outcomes and needs to be included in service provision, echoing World Health Organization initiatives to foster social connections.^47^ Notably, our data were collected before the coronavirus disease (COVID)-19 pandemic; the lonely profile likely represents a subgroup that may have faced disproportionate mental health and/or health care disruptions during pandemic-related isolation, suggesting our findings may underestimate loneliness prevalence post pandemic while still highlighting critical intervention targets. Effective loneliness interventions (eg, animal therapy, technologically facilitated social engagement, social support, and maladaptive social cognition treatment) among older adults could be considered, albeit some effects were moderate.^48^^,^^49^ For patients with coexisting diabetes and kidney failure, incorporating routine loneliness screening in clinical care with targeted strategies (eg, social skills training, structured group activities) could bolder social connectedness within dialysis settings. Stepped-up mental health supports may be required for those with persistent or severe distress, whereas resilience factors identified in the resilient profile could inform preventive approaches.

Moreover, the findings underline the need to empower patients with diabetes and kidney failure in their self-care abilities, particularly in diabetes management, diet adherence, and nutrition, to improve clinical prognosis. This aligns with World Health Organization’s advocacy of patient-centered self-care interventions to complement health systems in tackling the challenges of chronic conditions more effectively.^50^ The confirmation of mediation role of diabetes self-care further indicates that it may be a key target for interventions to alleviate the risk of hospitalization for patients experiencing loneliness. Evidence-based self-management support interventions for multimorbid populations, such as patient empowerment, social support, behavioral skill training, and care coordination, should be tailored to align with individual health literacy, physical function, and treatment goals and preferences to maximize effectiveness.

Our study provides a thorough assessment of psychosocial distress and self-care functions within a vulnerable population of patients with diabetes and kidney failure. Unlike traditional methods focusing on individual mood indicators, we used latent profile analysis to provide a holistic view of how multiple psychosocial risks coalesce to affect patient prognosis. Other methodological strengths include the control of significant clinical confounders and the use of mediation analysis that could accommodate different types of outcome variables, further increasing the reliability of our findings. In addition, using a racially diverse cohort undergoing detailed clinical evaluations with prospective follow-up adds robustness to our prognostic modeling.

Nevertheless, study limitations should be noted. First, we relied on self-reported measures for psychosocial distress and self-care behaviors, which may be subject to reporting bias. These biases may have attenuated observed associations, particularly if patients underreported stigmatizing symptoms (eg, depression) or overestimated self-care. Second, psychosocial distress and self-care data were collected at baseline, limiting the causal inference between these variables and the interpretation of mediation findings. Psychosocial distress may impair self-care behaviors, whereas worse self-care may lead to frustration and exacerbate mental health vulnerabilities. In addition, the psychosocial assessment occurred postdiagnosis, making it difficult to determine whether these patterns reflect pre-existing conditions or psychological reactions to kidney failure progression and its treatment burdens. This distinction has clinical significance, as premorbid conditions might warrant psychiatric interventions, whereas distress reactions may be more amenable to targeted coping interventions. Longitudinal studies tracking psychological trajectories from prediagnosis to postdiagnosis phases are needed to clarify these dynamics. Third, the sample was derived from an organization that serves socioeconomically disadvantaged groups in Singapore with a high burden of comorbid conditions, which may affect the generalizability of findings to other settings and populations. Furthermore, using administrative databases restricts our ability to differentiate between routine and emergency hospitalization events. Future research with larger sample sizes and extended observation periods should elucidate these associations and validate the findings across diverse populations and settings.

In conclusion, this study unraveled heightened hospitalization risks among patients with diabetes and kidney failure exhibiting lonely and overwhelmed psychosocial burden profiles relative to the resilient group. Furthermore, diabetes self-care acted as a pathway in explaining the excessive hospitalization risk associated with loneliness. These findings underscore the importance of incorporating comprehensive psychosocial screening and tailored interventions into clinical practice, particularly the need to address loneliness alongside traditional concerns of depression and anxiety. Psychosocial support and self-care interventions should be implemented to improve the prognosis of patients with diabetes and kidney failure.
