# Supplementary material for: Prognostication of Mental Health Risk Clusters on Hospitalization and Mortality in Patients With Coexisting Diabetes and Kidney Failure: The Hidden Burden of Loneliness
Source: Kidney Med. 2025 Sep 11;7(11):101099. doi: 10.1016/j.xkme.2025.101099 (PMC12597264; doi:10.1016/j.xkme.2025.101099)
Supplement: Supplementary File (PDF) — Figures S1, S2. [file mmc1.pdf]

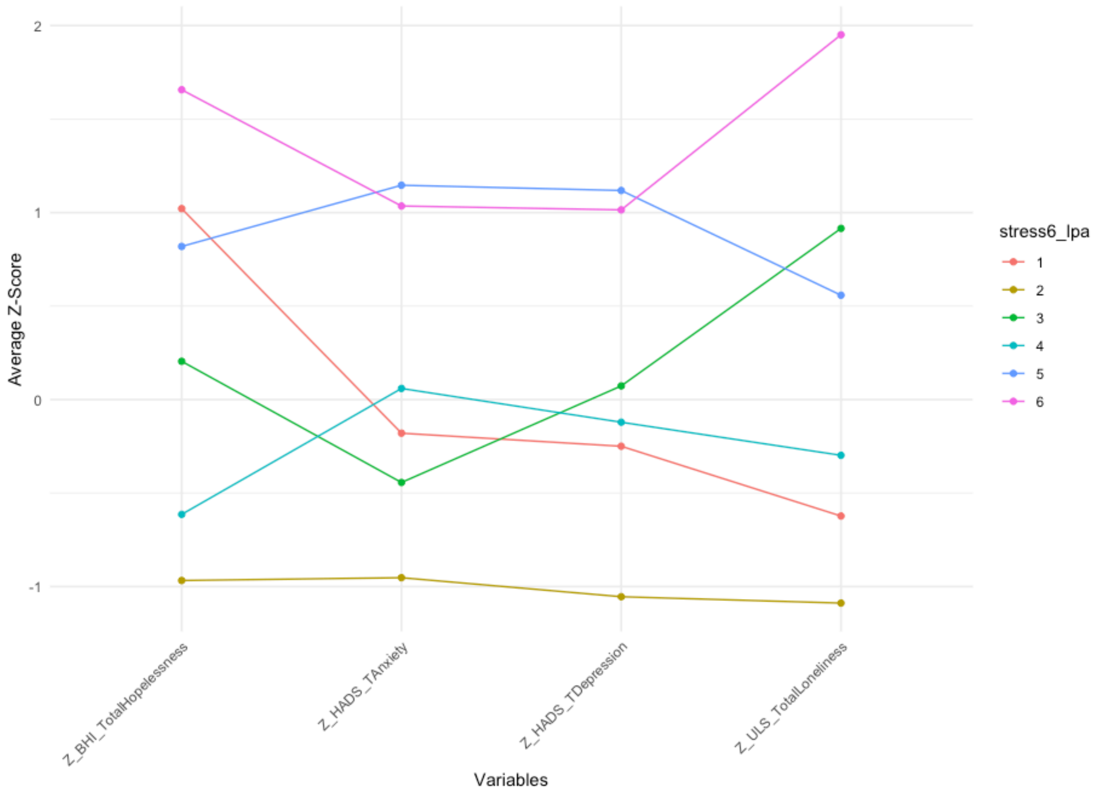

Figure S1. Description and standardized mean values of psychosocial indicators across profiles for the six-profile solution

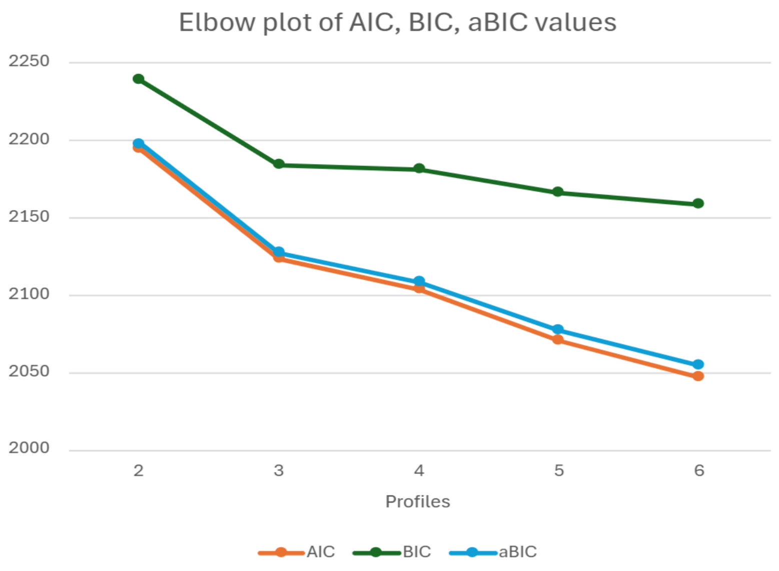

Figure S2. Elbow plots for the AIC, BIC, and aBIC indices
